# Genome-Wide Association Mapping for Tomato Volatiles Positively Contributing to Tomato Flavor

**DOI:** 10.3389/fpls.2015.01042

**Published:** 2015-11-27

**Authors:** Jing Zhang, Jiantao Zhao, Yao Xu, Jing Liang, Peipei Chang, Fei Yan, Mingjun Li, Yan Liang, Zhirong Zou

**Affiliations:** ^1^State Key Laboratory of Crop Stress Biology for Arid Areas, College of Horticulture, Northwest A&F UniversityYangling, China; ^2^Key Laboratory of Protected Horticultural Engineering in Northwest, Ministry of AgricultureYangling, China; ^3^College of Forestry, Northwest A&F UniversityYangling, China; ^4^Shaanxi Jinpeng Seed Industry Co. Ltd.Yangling, China

**Keywords:** tomato, volatile, genome-wide association study, flavor, quantitative trait loci

## Abstract

Tomato volatiles, mainly derived from essential nutrients and health-promoting precursors, affect tomato flavor. Taste volatiles present a major challenge for flavor improvement and quality breeding. In this study, we performed genome-wide association studies (GWAS) to investigate potential chromosome regions associated with the tomato flavor volatiles. We observed significant variation (1200x) among the selected 28 most important volatiles in tomato based on their concentration and odor threshold importance across our sampled accessions. Using 174 tomato accessions, GWAS identified 125 significant associations (*P* < 0.005) among 182 SSR markers and 28 volatiles (27 volatiles with at least one significant association). Several significant associations were co-localized in previously identified quantitative trait loci (QTL). This result provides new potential candidate loci affecting the metabolism of several volatiles.

## Introduction

The perception of the tomato flavor, including aroma and taste, is a result of the interaction of sugars, acids, and many aromatic volatile compounds (Goff and Klee, 2006; Klee and Tieman, 2013). Plants produce a large variety of fruit flavor volatiles. These volatiles are defining elements of the distinct flavors of fruits and are mainly derived from essential compounds, including amino acids, fatty acids, carotenoids (Goff and Klee, 2006; Klee and Tieman, 2013). However, selecting for the quality of fruit aroma has never been a high priority for plant breeders (Goff and Klee, 2006; El Hadi et al., 2013). In fact, research that is focused on improving fruit size, yield, quality, and shelf life has often led to unintended, negative outcome for both aroma and, consequently, flavor (Kader et al., 1977; Ratanachinakorn et al., 1997).

The effect of a volatile on flavor perception is determined by its concentration and perceptible aroma, or odor threshold. As few as 20 of more than 400 volatiles in tomatoes have sufficient concentrations and odor thresholds to contribute to tomato flavor (Baldwin et al., 2000). However, the biosynthetic pathways and regulatory networks are known for volatiles of the greatest economic importance, such as the 20 volatiles aforementioned (Klee, 2010; Klee and Tieman, 2013). Important genes that regulate volatiles can be broadly subdivided into two classes: genes encoding enzymes responsible for synthesis of the end products and genes encoding factors that regulate the pathway (Klee, 2010). The regulation of the pathways of most volatiles is very poorly understood. In fact, even our knowledge on the 20 most important volatiles in tomato is limited.

The genetic and molecular bases of volatiles are also poorly understood. This is primarily due to the polygenic nature and biochemical complexity of flavor/aroma traits and our limited ability to quantify those volatiles (Klee and Tieman, 2013). Improvements in quantification techniques, as well as the capacity for high-throughput genotyping (Agarwal et al., 2008), provide the basis for quantitative trait locus (QTL) analysis of aroma components. Such QTL studies of aroma have already been performed with apple (Zini et al., 2005), grape (Doligez et al., 2006; Obando-Ulloa et al., 2010), and melon (Obando-Ulloa et al., 2010). In tomato, more than 100 quantitative trait loci (QTLs) affecting volatiles and their precursors have been identified (Saliba-Colombani et al., 2001; Causse et al., 2004; Schauer et al., 2006; Tieman et al., 2006; Mathieu et al., 2008; Zanor et al., 2009). Some QTLs specifically alter single volatiles while others can affect several related or even unrelated volatiles (Mathieu et al., 2008).

Genome-wide association studies (GWAS) is a method for mapping the loci responsible for natural variations in a target phenotype (Saidou et al., 2014; Matsuda et al., 2015; Pers et al., 2015). It is based on the identification of significantly associated genetic polymorphisms in a large population (Brachi et al., 2011). GWAS is a reliable, preliminary approach for identifying the locations of QTLs and has been conducted on major fruit quality traits in tomato (Mazzucato et al., 2008; Ranc et al., 2012; Xu et al., 2013; Ruggieri et al., 2014; Zhang et al., 2015). However, few association mapping studies have been performed on QTLs for the main flavor-enhancing volatiles in tomatoes or other crop plants (Kumar et al., 2015). In this study, we performed a GWAS to locate QTLs for flavor-affecting volatiles in tomato. In particular, we detected volatiles in a collection of 174 diverse tomato accessions using gas chromatography-mass spectrometry (GC/MS). We also detected a large number of loci to link their volatile composition with their genotypes. Our results confirmed some previous volatile QTLs, and we identified some chromosome regions that could be important in controlling volatile metabolism.

## Materials and methods

### Plant materials

The tomato diversity panel consisted of 174 tomato accessions comprised of 123 cherry tomato accessions (*Solanum lycopersicum* var. *cerasiforme*) and 51 large-fruit cultivars (*S. lycopersicum*) (Table S1). All accessions were grown during the springs of 2013 and 2014 in a completely randomized block design with three replicates (10 plants per replicate), at the research greenhouse of the Tomato Research Group (34° 24_N, 108° 07_E) according to standard agronomic practices (Zhang et al., 2015).

### Volatile determinations

We performed analyses of fruit volatiles as described in Tikunov et al. (2005), with minor modifications. We combined all red, ripe fruit produced on the 10 accessions representing each horticultural type and immediately placed them in liquid nitrogen. We then kept them at −80°C. We transferred 15 g of the finely powdered tomato samples into a 40 mL Teflon cap vial (Thermo Fisher Scientific) with 5 g of NaCl. Ten microliters of 2-octanone (0.125 mg/mL in ethyl alcohol) were then added as an internal standard. We then sealed the vials using a silicone/PTFE septum and a magnetic cap. The closed vials were agitated (500 rpm) and sonicated for 10 min, and incubated at 50°C for 10 min prior to HS-SPME-GC-MS analysis. We performed three independent reactions for each sample. We extracted headspace volatiles by exposing each sample to a 75 μm carboxen-polydimethylsiloxane SPME fiber (Supelco, USA) for 30 min under continuous agitation (500 rpm) and heating at 40°C. The fiber was then inserted into an ISQ GC-MS (Thermo Scientific instruments, USA) injection port and the volatiles were desorbed for 3 min at 250°C. We performed chromatography on an HP-INNOWAX column (60 m × 0.25 mm × 0.25 μm) with helium as the carrier gas, at a constant flow of 1.0 mL min^−1^. The temperature of both the GC interface and MS source was 230°C. The GC temperature program began at 40°C (2.5 min), and then was raised to 160°C (5°C min^−1^) and to 230°C (10°C min^−1^) before being held at 230°C for 5 min. The total run time, including oven cooling, was 40 min. Mass spectra were evaluated in the 35–450 m/z range at a scanning speed of 70 scans s^−1^ and an ionization energy of 70 eV. We processed the raw data obtained from GC-MS with Xcalibur and AMDIS software and identified the volatile compounds on the basis of the NIST/EPA/NIH Mass Spectral Library (NIST 2008) and Wiley Registry of Mass Spectral Data 8th edition. The retention index (RI) was calculated with a homologous series of n-alkanes (C7–C30, Sigma-Aldrich). The volatiles were semi-quantified according to the method of Baek and Cadwallader (1996) and Hopfer et al. (2012). We calculated the relative concentration of each volatile using the ratio of the areas of the target peak and the internal standard (2-octanone, 0.125 mg/mL, 10 μL) in a total ion current chromatogram with the following equation:

$$\text{​​Relative concentration}=\left[\frac{\text{Peak area of particular compound}}{\text{Peak area of internal standard }(\text{IS})}\right]\times \text{Concentration of IS}$$

We used the relative concentrations to compare the differences in volatile profiles among the 174 accessions.

### Genotyping

We extracted DNA from the 174 tomato accessions from fresh leaf tissue following the method of Fulton et al. (1995). All SSR markers were mainly selected from the SOL Genomics Network (http://solgenomics.wur.nl/) and the VegMarks database (http://vegmarks.nivot.affrc.go.jp/). We used the protocol of Sun et al. (2012) to amplify the markers. Only markers with minor allele frequency (MAF) > 0.05 were genotyped with the whole accessions (Zhang et al., 2015). Finally, we selected a set of 182 polymorphic simple sequence repeat (SSR) for further association mapping (Zhang et al., 2015).

### Population structure

We used the above set of 182 SSR markers to estimate the population structure of the 174 tomato accessions via STRUCTURE 2.3.3 software (Pritchard et al., 2000). We set the number of hypothetical subpopulations (K) at 2–10 in order to evaluate the population structure with an admixture model. We performed 200,000 replicates of the Markov Chain Monte Carlo with a burn-in length of 100,000. We used Evanno transformation method to infer the optimal K of populations (Evanno et al., 2005). The kinship matrix was calculated via SPAGeDi software (Hardy and Vekemans, 2002). We set the diagonal of the matrix to two and negatives values to zero (Yu et al., 2005).

### Association mapping

Decay of LD and the corresponding significance level (*P*-value) were calculated using TASSEL 2.1 software (Bradbury et al., 2007). We also calculated associations between volatiles and SSR markers using TASSEL 2.1 software (Bradbury et al., 2007). Mixed linear model (MLM) was used in order to reduce false positive associations. We used *P* < 0.005 as the value to detect associations. We also took *P* < 0.0003 as the significant value to reduce false positive associations. The amount of phenotypic variation explained by each marker was estimated by *R*^2^.

### Statistics

We used either the SAS 8.1 program (SAS institute, Cary, NC) or the R statistical Software (http://www.r-project.org) version 3.0.2 for statistical analyses. We replaced the values of zero (undetectable) for all volatiles by the smallest non-zero value in the whole dataset (Mathieu et al., 2008). Then, we log_2_-transformed the volatile quantity values (ng g^−1^ fresh weight h^−1^) before performing a Two-way ANOVA analysis for all traits. The resulting raw *P*-values were also corrected for multiple tests using the Benjamini and Hochberg FDR test (Benjamini and Hochberg, 1995). We estimated genetic variance, genetic by environment interaction variance, technical variance, heritability values according to the method of Xu et al. (2013). We developed correlation heat map via HemI 1.0., based on the analysis result among volatiles and accessions.

## Results

### Variation in volatiles

Of the more than 400 volatiles reported in tomato, fewer than 20 have been predicted to contribute to the unique tomato flavor based on the concentrations and odor thresholds (Buttery et al., 1987; Goff and Klee, 2006; Klee, 2010; Tieman et al., 2012). Here, we further investigated 28 volatiles that are in sufficient quantities to impact the tomato flavor. We found large variations of up to 1200 x in volatile contents across all of the sampled accessions (Table 1).

**Table 1:** **Volatile variation within the 174 tomato accessions (ng/g fresh weight/hr)**.

**Compound**	**Max**	**Min**	**Fold difference**	**Mean**	**SD**	***H^2^***
1-Penten-3-ol	33.73	1.37	25	7.35	6.66	0.71
3-Methylbutanol	224.42	1.13	199	13.17	25.30	0.58
1-Pentanol	139.68	1.29	109	11.22	15.14	0.54
1-Hexanol	937.23	1.18	797	164.77	187.10	0.76
(E)-2-Hexen-1-ol	85.68	1.16	74	14.53	14.97	0.66
(Z)-3-Hexen-1-ol	557.15	1.44	387	77.39	95.52	0.67
6-Methyl-5-hepten-2-ol	145.66	1.26	115	19.33	26.22	0.39
2-Phenylethanol	13.58	0.11	119	2.32	3.13	0.44
Methyl salicylate	2.16	0.12	18	0.52	0.45	0.30
Beta-ionone	0.98	0.11	9	0.43	0.25	0.41
1-Penten-3-one	5.44	0.12	44	1.03	1.16	0.56
6-Methyl-5-hepten-2-one	73.34	0.14	540	10.39	13.06	0.65
Geranylacetone	54.62	0.11	476	5.07	6.40	0.42
Eugenol	44.35	1.19	37	18.04	12.38	0.36
2-Isobutylthiazole	78.94	0.13	590	6.87	10.96	0.55
Limonene	43.12	1.55	28	11.25	10.55	0.47
2-Pentylfuran	91.84	1.13	81	24.56	19.94	0.40
Beta-cyclocitral	89.26	1.12	80	13.73	14.61	0.58
Geranial	355.69	0.29	1238	62.63	63.18	0.64
Neral	132.50	2.58	51	26.44	33.93	0.68
(Z)-3-Hexenal	524.47	1.16	451	60.15	96.12	0.75
(E)-2-Hexenal	131.58	1.44	92	12.38	16.80	0.71
Hexanal	666.31	1.44	463	79.14	114.64	0.69
(Z)-2-Heptenal	98.58	1.38	72	15.39	14.43	0.52
(E,E)-2,4-Heptadienal	75.54	1.20	63	10.62	12.87	0.60
(E,E)-2,4-Nonadienal	91.25	1.20	76	13.92	16.21	0.53
Beta-damascenone	13.15	1.22	11	4.98	3.35	0.32
(E)-2-Pentenal	30.56	1.69	18	9.71	8.67	0.54

*Max, Maximum; Min, minimum; SD, standard deviation; H^2^, heritability*.

We calculated heritability values for all 28 volatiles based on 2 years of phenotypic characterization. Of these volatiles, nine had a value lower than 0.5 (Table 1). Therefore, volatile values in 2013 and 2014 were evaluated separately for further genome-wide association analyses.

**Figure 1:**
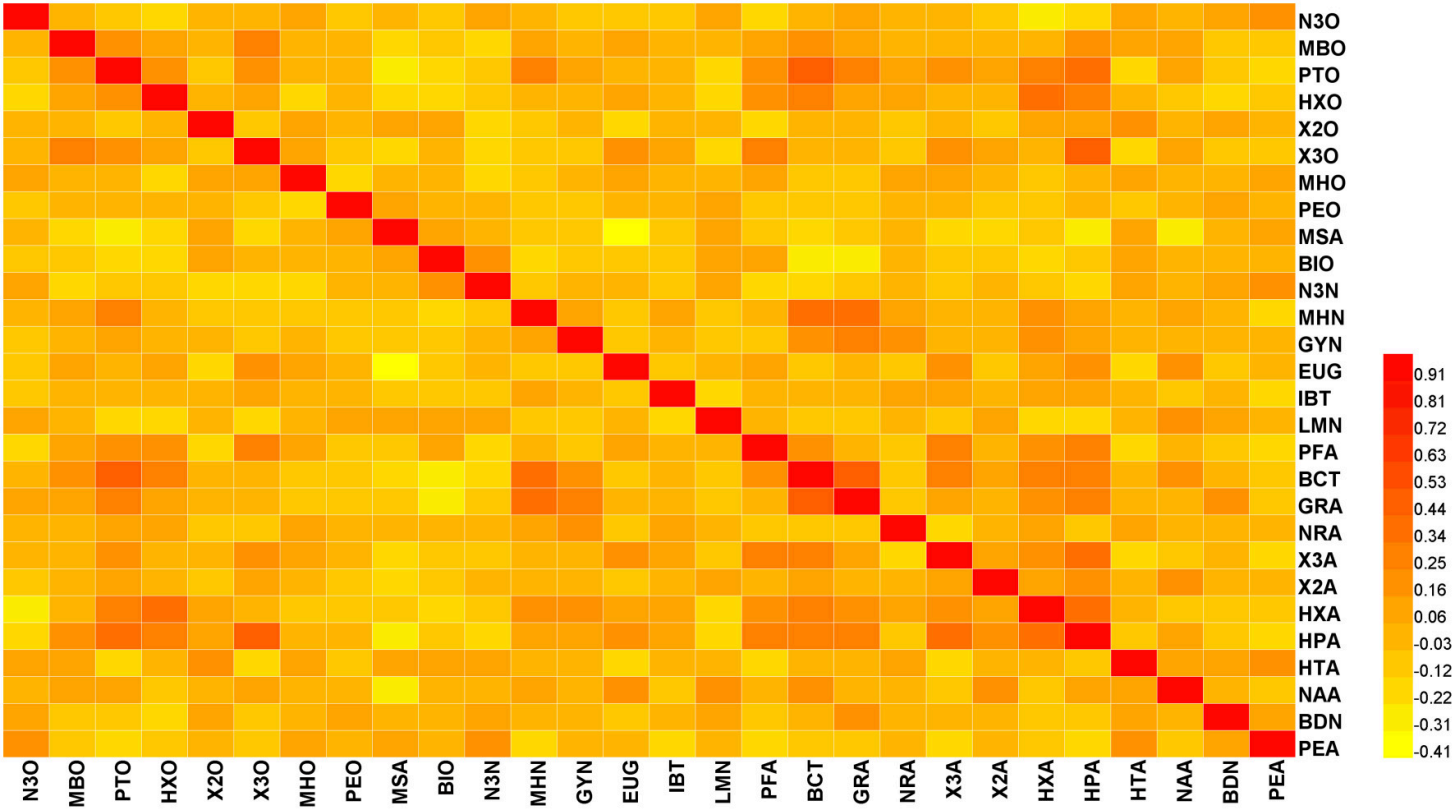
**Heat map showing the correlation analysis between 28 volatiles in the 174 tomato accessions**. Regions in red and yellow indicate positive or negative correlations between traits, respectively (The complete data is available in Table S2).

Pearson correlation coefficients (*r*) among the 28 volatiles were relatively low, based on the mean value of 2 years data (the springs of 2013 and 2014) (lower than 0.5) (Figure 1). However, we observed significant coefficients among some volatiles (Table S2). For instance, beta-cyclocitral and (Z)-2-heptenal were positively correlated with 1-pentanol (*r* = 0.464 and 0.417, respectively). In addition, methyl salicylate was negatively correlated with eugenol, (Z)-2-heptenal, and (f)-2,4-heptadienal (*r* = −0.406, −0.266, and −0.241, respectively).

**Figure 2:**
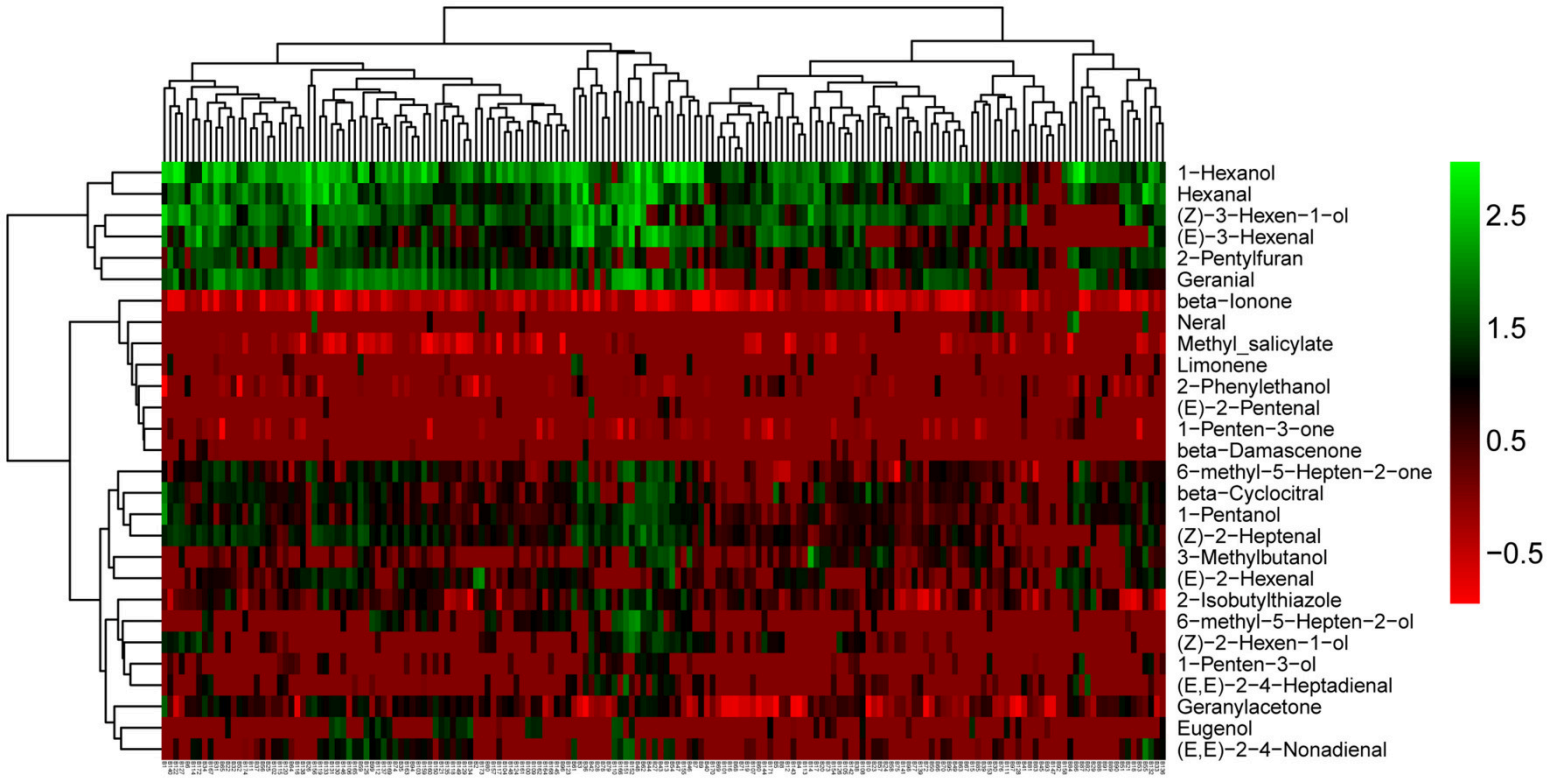
**Cluster analyses of the 28 selected volatiles among the whole accessions**. The names of volatiles (right) and the accession codes (bottom) are shown (The accession codes can be seen in Table S1).

We observed that volatiles derived from the same pathway had a tendency to cluster together (Figure 2). In addition, we observed large variation among the 28 volatiles in the cluster analysis for all of the accessions (Figure 2). The largest variations were mainly observed for 1-hexenol, hexanal, (Z)-3-hexen-1-ol, (Z)-3-hexenal. Additionally, these four volatiles were all linolenic acid-derived flavor molecules and were closely clustered. (Z)-3-hexen-1-ol can be derived from (Z)-3-hexenal by alcohol dehydrogenase (ADH). Similarly, 1-hexenol can also be derived from hexanal via ADH. Volatiles, structures, identification and their precursors of the selected 28 volatiles used in this research are presented in Table S3.

### Molecular polymorphism

The 174 sampled accessions were genotyped with 182 SSRs. We selected only markers with MAF > 5% for association mapping (Zhang et al., 2015). The distributions of the MAF were different among the three groups of accessions. The average MAF for all of the accessions, *S. l. cerasiforme* accessions and *S. lycopersicum* accessions has been described by Zhang et al. (2015).

**Figure 3:**
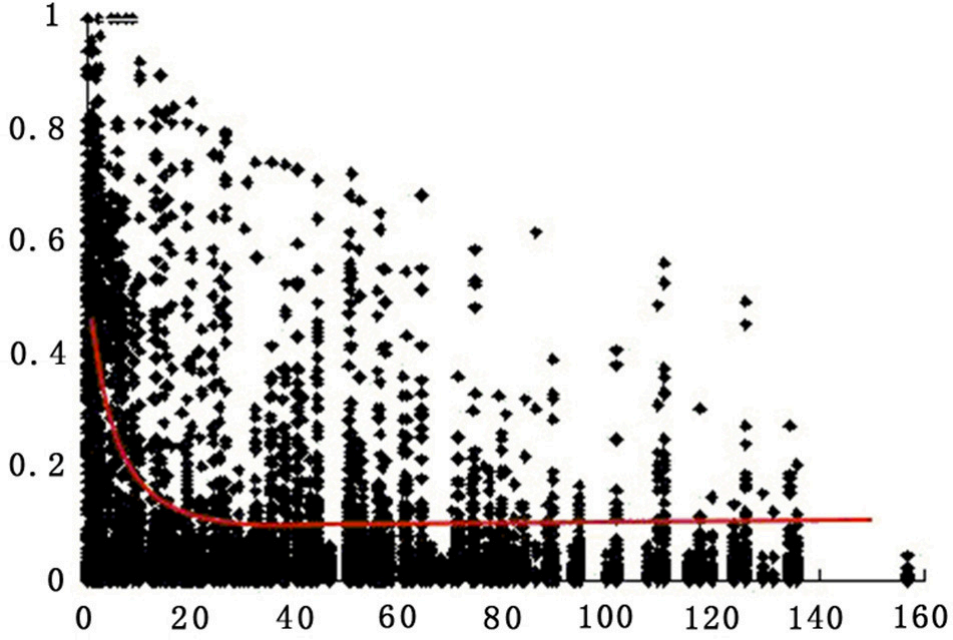
**Estimates of LD (*r*^2^) over genetic distance on all chromosomes for all 174 tomato accessions**. Only polymorphic sites with MAF >0.05 are indicated (see Materials and Methods). Plot of *r*^2^ over genetic distance if fitted by non-linear regression (red curve).

LD decay was analyzed for all markers on all chromosomes for the 174 accessions. Pairwise *r*^2^ was plotted according to the chromosome genetic distance between loci. Non-linear regression was fitted to the decay of LD over genetic distance. LD on the whole genome for all accessions extended on average over 8 cM for *r*^2^ = 0.2 (Figure 3).

### Population structure

We assessed population structure of the 174 tomato accession using STRUCTURE 2.3.3 software with 182 SSR markers. We found an optimal *K* = 2 inferred according to Evanno method (Evanno et al., 2005). The inferred population was congruent with *S. l. cerasiforme* and *S. Lycopersicum* accessions, respectively (Zhang et al., 2015).

### Genome-wide association analysis

In order to reduce false positive associations, we used the K+Q model (kinship matrix and genetic structure) to detect associations between the selected 28 volatiles and 182 SSR markers. We analyzed the phenotypic data in 2013 and 2014 separately. In total, we detected 125 marker-trait associations (MTAs) on 28 selected volatiles in 2013 and 2014 (*P* < 0.005) (Table 2). Among these, 52 MTAs were detected in both years. Twenty-nine of them are significant associations (*P* < 0.0003). In 2013, 2014, we detected 82, 95 MTAs, respectively. We detected at least one MTA for each volatile. The only exception is for eugenol and we detected no MTAs for this volatile. The most significant MTA for all volatiles in both year was detected on 6-methyl-5-hepten-2-one. This MTA was detected on TES344 on chromosome 11 (Chr11), explaining 36.38, 33.25% of the phenotypic varation, in 2013 and 2014, respectively. The other most significant MTA was detected for (E)-2-hexen-1-ol. This MTA was detected on SSR 287 (Chr2), explaining 44.51, 42.13% of the phenotypic variation in 2013 and 2014, respectively.

**Table 2:** **Marker-trait associations for 28 volatiles greatly affecting tomato flavor estimated with K+Q (MLM) model on 174 tomato accessions (only those where *P* < 0.005 are listed)**.

**Compound**	**Locus**	**Chr^a^**	**Position^b^**	**2013**		**2014**	
				**Corrected *P*-value^c^**	***R*^2^**	**Corrected *P*-value**	***R*^2^**
1-Penten-3-ol	TGS925	7	41.54	ns	–	0.0028	0.0562
	TES246	11	63.67	0.0044	0.043	ns	–
3-Methylbutanol	SSR92	1	0	4.07E-12	0.3069	6.87E-09	0.2171
	SSR26	2	77.5	0.0043	0.0487	1.12E-05	0.1213
	LEgata001	3	NG^d^	4.59E-04	0.1049	ns	–
	SSR320	3	107.3	ns	–	1.82E-04	0.1196
	SSR150	4	115	6.20E-06	0.147	3.18E-05	0.1078
	SSR325	5	18.5	ns	–	0.0026	0.0667
	SSR13	5	28	4.90E-12	0.3225	5.52E-10	0.2176
	TGS266	6	32.26	ns	–	0.0011	0.066
	TES1966	10	88.92	2.13E-04	0.0493	0.0011	0.1002
	TES1449	11	73.38	4.66E-10	0.191	1.12E-11	22.13
1-Pentanol	TES291	1	62.98	ns	–	0.0021	0.0539
	SSR598	2	78	0.0041	0.0572	ns	–
	SSR320	3	107.3	ns	–	0.0047	0.0657
	TES835	3	123.55	ns	–	0.0014	0.0891
	SSR133	4	30.6	8.73E-09	0.2672	7.45E-10	0.2534
	SSR345	12	72.5	6.20E-09	0.2284	3.78E-10	0.2215
1-Hexanol	SSR306	4	56	0.0034	0.0677	ns	–
	TES623	9	83.56	0.0016	0.0357	0.0035	0.0356
	TGS551	10	32.33	0.0043	0.0318	ns	–
(E)-2-Hexen-1-ol	SSR287	2	107	1.61E-24	0.4451	3.92E-24	0.4213
	TGS639	5	74.02	ns	–	0.0032	0.052
	TGS354	8	30.65	0.0016	0.0581	ns	–
	TGS607	8	37.89	0.0050	0.0413	ns	–
	TGS560	9	78.87	ns	–	0.0026	0.1222
	TGS738	10	1.6	0.0045	0.0424	ns	–
(Z)-3-Hexen-1-ol	SSR188	4	135.5	6.72E-04	0.0789	3.28E-04	0.0838
	TES99	5	125.75	0.0011	0.0255	ns	–
	TES628	6	2.23	1.88E-04	0.0789	4.28E-04	0.0759
	TES1427	8	16.59	0.0046	0.033	ns	–
	TGS607	8	37.89	0.0049	0.0307	0.0047	0.0356
	TES40	10	56.34	0.0044	0.0372	ns	–
6-Methyl-5-hepten-2-ol	SSR92	1	0	ns	–	1.21E-04	0.0079
	SSR20	2	58	3.53E-06	0.1458	6.35E-04	0.1011
	TES88	2	156.68	9.61E-04	0.0514	ns	–
	TGS827	3	4.42	1.89E-04	0.0856	ns	–
	SSR128	6	35	0.0021	0.1056	9.35E-04	0.0642
	TOM166	9	3.1	1.37E-07	0.1969	0.0049	0.0785
2-Phenylethanol	TES835	3	123.55	ns	–	0.0022	0.0844
	TES945	6	87.82	ns	–	9.35E-04	0.0642
	TES1521	7	79.06	3.84E-05	0.1059	7.87E-04	0.062
	TGS868	8	6.57	0.0018	0.1079	0.0016	0.0576
	TOM166	9	3.1	3.18E-04	0.0919	0.0044	0.0608
Methyl salicylate	TGS266	6	32.26	ns	–	2.53E-04	0.0657
	TGS1380	9	49.16	ns	–	0.0011	0.0511
	TES718	10	83.6	ns	–	0.0037	0.0467
Beta-ionone	SSR598	2	78	ns	–	5.28E-04	0.0701
	SSR45	7	75.5	ns	–	3.12E-05	0.0694
	TES1407	10	42.91	0.0050	0.0452	ns	–
	TES718	10	83.6	8.00E-04	0.0705	0.0018	0.033
	TES124	11	73.69	0.0049	0.0613	ns	–
1-Penten-3-one	TES363	5	1.54	ns	–	4.33E-04	0.0808
	TES628	6	2.23	ns	–	0.0030	0.0675
	TGS925	7	41.54	ns	–	9.03E-04	0.0734
	TES1427	8	16.59	0.0036	0.0513	0.0018	0.0536
6-Methyl-5-hepten-2-one	TGS827	3	4.42	0.0043	0.0486	ns	–
	SSR188	4	135.5	4.50E-07	0.1789	5.71E-06	0.1542
	TES36	9	0.73	0.0011	0.1202	ns	–
	TOM166	9	3.1	0.0010	0.0979	5.53E-04	0.1023
	TES344	11	51.42	1.51E-26	0.3638	1.84E-24	0.3325
Geranylacetone	SSR133	4	30.6	0.0016	0.1339	0.0012	0.143
	SSR325	5	18.5	ns	–	0.0032	0.0676
	SSR13	5	28	ns	–	8.13E-04	0.0423
	TES945	6	87.82	4.36E-04	0.0786	ns	–
	SSR122	6	101	1.19E-06	0.1692	1.69E-05	0.1565
	TGS2911	6	93.92	2.07E-04	0.0971	0.0037	0.054
	TES520	7	0.04	3.11E-04	0.0819	ns	–
	TGS2132	8	19.52	0.0018	0.0589	ns	–
	SSR142	9	16.5	2.27E-06	0.1902	1.09E-05	0.1765
	SSR110	9	55.7	2.72E-05	0.1439	4.67E-05	0.1322
2-Isobutylthiazole	SSR287	2	107	ns	–	3.21E-04	0.1052
	TES1179	6	40.18	ns	–	0.0013	0.1185
	SSR344	8	4	6.88E-04	0.1279	9.35E-04	0.0636
	TGS738	10	1.6	ns	–	0.0015	0.635
Limonene	TES816	6	67.53	1.11E-04	0.0955	0.0023	0.0741
	SSR142	9	16.5	ns	–	6.87E-04	0.0254
	TGS560	9	78.87	ns	–	5.28E-04	0.1529
2-Pentylfuran	TES1179	6	40.18	ns	–	0.0026	0.0782
Beta-cyclocitral	TGS1548	2	77.52	6.97E-06	0.143	5.51E-05	0.149
	TES1276	2	82.99	8.43E-06	0.1406	7.20E-05	0.1532
	TGS364	5	46.19	0.0043	0.0629	ns	–
	SSR128	6	35	6.75E-05	0.1561	8.14E-04	0.067
	TGS1973	6	80.07	0.0047	0.0632	2.41E-04	0.086
	TGS1629	9	6.41	ns	–	0.0014	0.0067
Geranial	SSR150	4	115	0.0041	0.0527	5.05E-05	0.0345
	TES1179	6	40.18	ns	–	9.86E-04	0.0453
	TES520	7	0.04	3.09E-04	0.0659	ns	–
	TGS2132	8	19.52	3.96E-04	0.0623	0.0021	0.0583
	SSR142	9	16.5	0.0028	0.075	0.0025	0.0702
	SSR110	9	55.7	0.0043	0.0597	0.0030	0.0583
Neral	SSR92	1	0	ns	–	2.72E-04	0.1422
	TGS420	3	21.07	0.0011	0.0807	8.49E-04	0.932
	SSR276	7	18	3.25E-04	0.0884	ns	–
	TGS153	12	28.96	0.0050	0.0398	0.0037	0.0796
(Z)-3-Hexenal	TGS1032	9	30.1	ns	–	0.0032	0.0143
(E)-2-Hexenal	TGS827	3	4.42	ns	–	0.0039	0.0322
	SSR342	6	19.1	0.0024	0.0728	ns	–
Hexanal	TES287	4	69.72	ns	–	0.0011	0.0697
	TES36	9	4.22	ns	–	0.0017	0.0621
	TES1028	9	83.73	ns	–	0.0019	0.0587
(Z)-2-Heptenal	TES358	5	60.96	0.0031	0.0348	ns	–
	TGS467	6	42.82	1.45E-05	0.1021	5.28E-04	0.0794
	TES1899	6	70.98	7.84E-04	0.0437	ns	–
	TGS1032	9	30.1	1.10E-08	0.1876	7.01E-08	0.1625
	TGS1380	9	49.16	3.28E-06	0.1017	1.39E-06	0.1519
	TES340	9	49.98	3.28E-06	0.1017	1.39E-06	0.1517
	TES1246	9	54.71	1.84E-05	0.0731	4.05E-04	0.0845
	TGS1713	9	56.86	3.63E-06	0.102	7.35E-06	0.1518
	SSR80	11	16.8	0.0021	0.042	ns	–
(E,E)-2,4-Heptadienal	SSR266	1	32.7	ns	–	0.0019	0.0779
	SSR20	2	58	0.0031	0.0687	ns	–
	TES748	3	88.04	ns	–	0.0016	0.0625
	TES618	12	15.07	ns	–	8.10E-04	0.0646
(E,E)-2,4-Nonadienal	TGS639	5	74.02	ns	–	1.14E-04	0.0984
	TES628	6	2.23	ns	–	5.28E-04	0.0969
	TES36	9	0.73	0.0041	0.0974	0.0011	0.1318
Beta-damascenone	SSR133	4	30.6	0.0014	0.1372	7.18E-06	0.1045
	TES816	6	67.53	3.69E-06	0.1355	1.69E-05	0.1364
	SSR122	6	101	6.56E-06	0.1286	5.98E-05	0.065
	SSR142	9	16.5	ns	–	0.0024	0.0751
	SSR110	9	55.7	ns	–	0.0036	0.0631
(E)-2-Pentenal	SSR320	3	107.3	0.0038	0.0762	ns	–
	SSR188	4	135.5	0.00	0.0619	ns	–
	TES363	5	1.54	6.19E-04	0.0707	0.0041	0.0467

a *Chromosome*.

b *Genetic distance of the marker was not found in EXPEN2000 reference map (http://www.solgenomics.net)*.

c *P-values are corrected following the Benjamini and Hochberg (1995) procedure (see Materials and Methods)*.

d *Genetic distance of the marker was not found in EXPEN2000 reference map (http://www.solgenomics.net)*.

*ns, no significant; –, not given*.

### Carotenoid-derived volatiles

Of the 28 volatiles selected for association mapping, there are three important open chain carotenoid-derived volatiles, 6-methyl-5-hepten-2-one, 6-methyl-5-hepten-2-ol and geranylacetone. There are another three cyclic carotenoid-derived volatiles, including beta-ionone, beta-cyclocitral, and beta-damascenone. For 6-methyl-5-hepten-2-one, derived by oxidative cleavage of lycopene, five MTAs were detected. Among these, the associated marker TES344 (Chr11) showed the most significant association value (*P* = 1.51E-26, in 2013; *P* = 1.84E-24, in 2014) among all significant MTAs detected for 28 volatiles. For 6-methyl-5-hepten-2-ol, which is directly biochemically linked with 6-methyl-5-hepten-2-one via ADH, we detected six significant MTAs. Among these, three MTAs were detected in both years. The most significant associated marker was TOM166 (Chr3) in 2013, which explained about 19.69% of the total phenotypic variation. We also detected significant association on this marker in 2014. The significantly level is relatively lower, explaining 7.85% of the phenotypic variation. This marker was also significantly associated with 6-methyl-5-hepten-2-one, and explained approximately 9.79, 10.23% of the phenotypic variation, in 2013 and 2014, respectively. Marker TGS827 (Chr3) was also associated with 6-methyl-5-hepten-2-one and 6-methyl-5-hepten-2-ol, and explained 4.86 and 8.56% of the phenotypic variation in 2013, respectively. However, we observed no significant MTAs on both volatiles on this marker in 2014. For geranylacetone, we found 10 MTAs, and five of them were detected in both 2013 and 2014. The most significant of these was with marker SSR122 (Chr6), which explained 16.92, 15.65% of the phenotypic variation, respectively. The other most significant MTA of these was with SSR142 (Chr9). This MTA could explain 19.02, 17.65% of the phenotypic variation, in both years, respectively.

Significant MTAs were also observed for three cyclic carotenoid-derived volatiles, with five, six and five MTAs, respectively. The most significant MTA for beta-cyclocitral was detected on marker TGS1548 (Chr2), both in 2013 and 2014. This MTA explained 14.3, 14.9% of the phenotypic variation, respectively. The most significant association for beta-damascenone was detected on TES816 (Chr6), explaining 13.55, 13.64% of the phenotypic variation, in 2013 and 2014, respectively.

### Lipid-derived volatiles

Of the 28 volatiles in sufficient quantities to impact the tomato flavor, we discovered MTAs for 11 lipid-derived volatiles (Table 2). For (E)-2-hexen-1-ol, we found six MTAs. The most significantly associated marker was SSR287 (Chr2). This MTA represented one of the most significant MTAs for all 28 volatiles, and explained 44.51, 42.13% of the total phenotypic variation, in 2013 and 2014, respectively. Only two MTAs were detected for (E)-2-hexenal, with one MTA in each year, respectively. For 1-penten-3-one, four MTAs were detected either in 2013 or 2014. For 1-penten-3-ol, which is directly biochemically linked with 6-methyl-5-hepten-2-one via ADH, only two MTAs were detected. For 1-pentanol, six MTAs were detected and two had a high association value. The two associated markers were SSR345 (Chr12) and SSR133 (Chr4). The associated marker SSR345 explained 22.84, 22.15% of the phenotypic variation, in 2013, 2014, respectively. Marker SSR133 accounted for 26.72, 25.34% of the phenotypic variation, respectively. For (Z)-2-heptenal, we discovered nine significant MTAs, and six of them were detected both in 2013 and 2014. Among the nine MTAs, five of which were located on Chr9 in the region from 30.1 to 56.86 cM. The most significant associated marker was TGS1032, located at about 30.1 cM on Chr9. This MTA which explained 18.76, 16.25% of the phenotypic variation, in 2013, 2014, respectively.

### Amino acid-derived volatiles

For 3-methylbutanol, a leucine-derived flavor volatile, we found 10 MTAs. The two most significant MTAs involved with marker SSR92 (Chr1) and SSR13 (Chr5). These two MTAs responsible for 30.69 and 32.25% of the total phenotypic variation in 2013, respectively. In 2014, they accounted for 21.71, 21.76% of the total phenotypic variation. For 2-phenylethanol, a phenylalanine-derived volatile, we observed five MTAs in either 2013 and 2014. The most significant marker was TES1521 (Chr7), explaining 9.19, 6.2% of the phenotypic variation, respectively.

### Terpenoid-derived volatiles

The two most important terpenoid-derived volatiles in tomato are neral and geranial, which are primarily localized in tomato leaves and stems (Buttery and Ling, 1993). However, we still observed these two volatiles in fruits in many tomato accessions. We observed four MTAs for neral and six for geranial in either year. For neral, the most significant MTA was detected on SSR92 (Chr1) in 2014, explaining about 14% of the phenotypic variation. No significance was observed on this marker in 2013. For geranial, the most significant MTA was also detected in 2014. This MTA was detected on marker SSR150 (Chr4), accounting for 3.45% of the phenotypic variation.

## Discussion

Genome-wide association study (GWAS) is a useful tool to detect candidate loci responsible for the natural variations in a targeted phenotype. This tool can identify significant associations between polymorphic molecular markers and targeted traits in a large natural population (Weigel, 2012; Matsuda et al., 2015). However, many factors can impact the results of association mapping, including type and size of mapping population, targeted traits, number of environments and years for phenotypic evaluations and the type and genome coverage of molecular markers (Ruggieri et al., 2014). Thus, we used a large sample of cherry and large fruited tomato accessions and a MLM to reduce false positive associations in GWAS (Zhao et al., 2007; Bernardo, 2008). The population in our study composes 123 cherry tomato accessions and 51 large fruited accessions and we think the size of our collection was adequate for GWAS (Ranc et al., 2012; Xu et al., 2013; Ruggieri et al., 2014).

### Phenotypic and genetic diversity

This whole studied population composes 123 cherry tomato and 51 large fruited accessions, representing a large diversity (Table 1). We found that this population has a large phenotyic diversity, such as fruit weight, soluble solid content, and lycopene content, etc. (Zhang et al., 2015). The large variations of the selected 28 crucial volatiles up to 1200x confirmed this (Table 2). The studied population could be mainly divided into two subgroups, cherry and large-fruited (Zhang et al., 2015). The higher MAF value among the studied population confirmed that *S. l. cerasiforme* (cherry tomato) is a mosaic of *S. lycopersicum* (large fruited tomato) and *S. pimpinellifolium* (wild species) (Frary et al., 2000; Zhao et al., 2007; Ranc et al., 2008; Xu et al., 2013; Zhang et al., 2015). The linkage disequilibrium of the whole genome decays at about 8 cM, which is consistent with previous studies (van Berloo et al., 2008; Xu et al., 2013; Zhang et al., 2015). Therefore, using a large collection of cherry tomato accessions together with cultivated tomato accessions is useful to overcome the high linkage disequilibrium value of tomato genome (Xu et al., 2013; Zhang et al., 2015).

### Associations confirmed identified volatile QTLs

**Figure 4:**
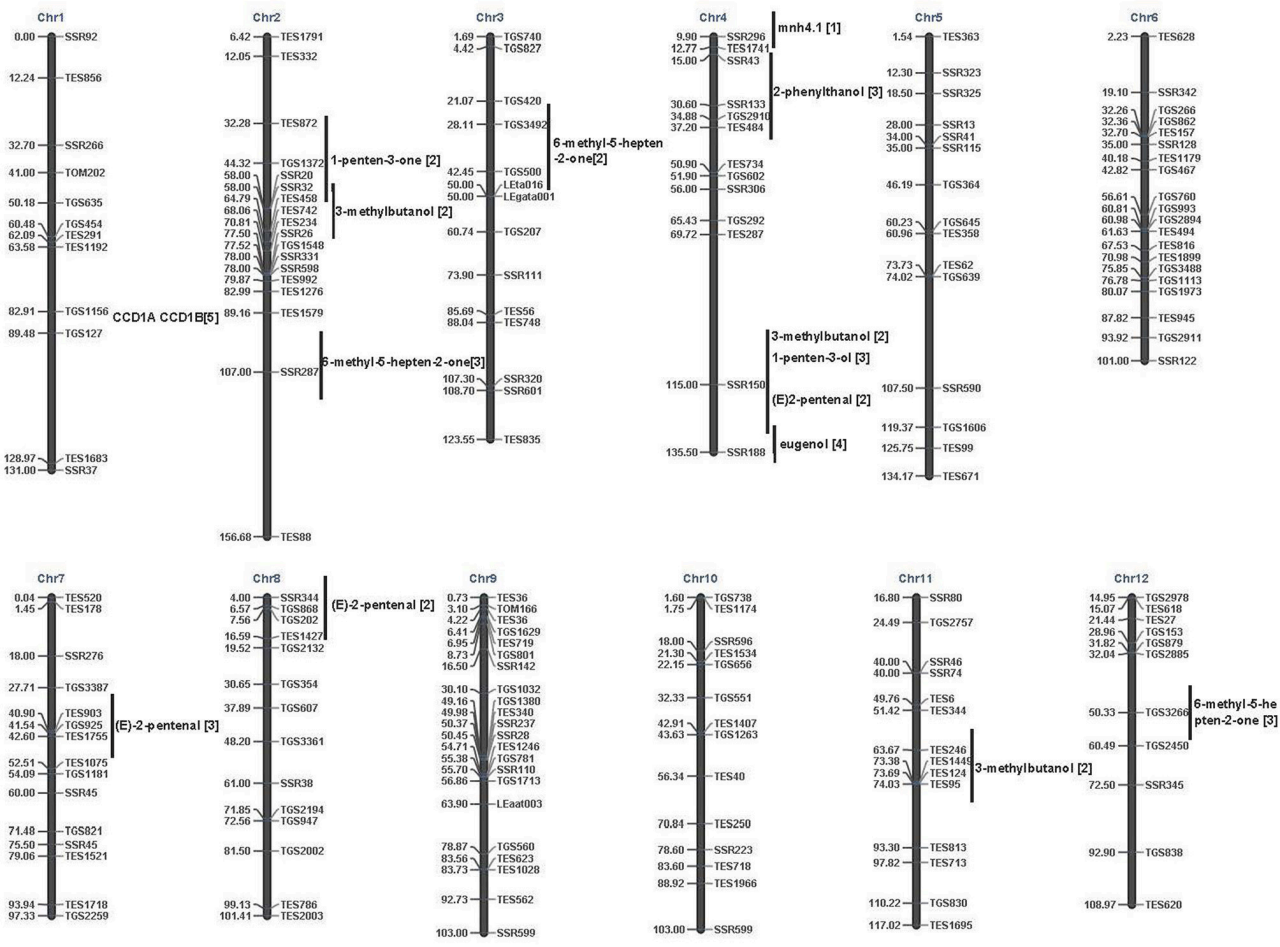
**Comparison of associations and QTLs identified by linkage mapping**. Horizontal line corresponds to the genetic location of associated marker **(right)** and the associated volatiles **(left)**. Vertical line is the approximate regions of the identified QTLs. QTLs identified by Saliba-Colombani et al. (2001) were indicated by [1] to the end of volatiles; QTLs identified by Tieman et al. (2006) were indicated by adding [2] to the end of volatiles; QTLs identified by Mathieu et al. (2008) were indicated by [3] to the end of volatiles; QTLs identified by Zanor et al. (2009) were indicated by [4] to the end of volatiles. CCD, carotenoid cleavage dioxygenases. CCD1A and CCD1B are two genes from the tomato genome data and were indicated by [5] to the end of genes. In Simkin et al. (2004), these two genes were listed as *LeCCD1A* and *LeCCD1B*.

The tomato is an excellent model for investigating the molecular basis of flavor using association mapping (Klee and Tieman, 2013). To date, few association mapping studies have focused on volatiles in major crops (Kumar et al., 2015). Here, we conducted GWAS between 28 most volatiles in tomato and SSR markers and found significant MTAs for most of the studied volatiles (Table 2). In tomato, over 50 QTLs affecting volatile levels have been identified, mainly using recombinant inbred lines (RIL) or introgression lines (IL) (Saliba-Colombani et al., 2001; Tieman et al., 2006; Mathieu et al., 2008; Zanor et al., 2009). However, the size of introgressed regions is large large (about 10–40 cM) (Zanor et al., 2009) and the results among prior studies differed. For example, prior studies have found different QTLs for the 6-emthyl-5-hepten-2-one, an important carotenoid-derived volatile. In particular, one major QTL *mhn4.1* impacting 6-emthyl-5-hepten-2-one was detected on chromosome 4 (Chr4) using an introgression line (Saliba-Colombani et al., 2001). However, two different QTLs were detected on 2A, 3C, and 12D in other IL populations (Tieman et al., 2006; Mathieu et al., 2008; Figure 4). In our research, we found six significant associations (*P* < 0.005) for 6-emthyl-5-hepten-2-one. Among them, the most significant associations were detected on Chr11 (TES344) and Chr4 (SSR188). These two associations were also found in the near region of two QTLs for 6-emthyl-5-hepten-2-one in a previous study by Tieman et al. (2006).

Twenty-five significant MTAs were detected for 15 volatiles on Chr4 (Table 2). The associations observed on Chr4 showed support for five previously-identified QTLs, including QTLs for3-methylbutanol and (E)-2-pentenal by Tieman et al. (2006); QTLs detected for 2-phenythanol and 1-penten-3-ol by Mathieu et al. (2008); and one QTL detected for eugenol by Zanor et al. (2009). However, only a few co-localized QTLs with the significant associations were observed on Chr4 (Figure 4). This could be mainly due to the limited volatiles sampled in previous studies and the limited molecular markers (Saliba-Colombani et al., 2001; Tieman et al., 2006; Mathieu et al., 2008; Zanor et al., 2009). In fact, among the28 volatiles that we sampled, only about 10 volatiles were mentioned in previous studies. Based on previous GWAS on tomato, the LD of tomato genome decayed at approximately 5–20 cM (Mazzucato et al., 2008; van Berloo et al., 2008; Ranc et al., 2012; Xu et al., 2013). Therefore, a 5–10 cM genome coverage should be enough to detect positive associations, especially by using SSRs. Our research revealed more polymorphic loci impacting tomato volatile profiles. However, among the detected 125 MTAs, only 52 of them were detected in both years. The overall significance value is still relatively low. A 5.2 cM genome coverage could detect positive associations. Still, more markers are needed to have a higher genome resolution to detected more candidate QTLs or genes. In addition, the tomato genome data has been available. In order to conduct more efficient association mapping, marker assisted selection and fine mapping of QTLs, high-throughput SNP chips via conducting re-sequencing on the core tomato accessions would greatly promote our further research.

### Volatile biosynthesis pathways

**Figure 5:**
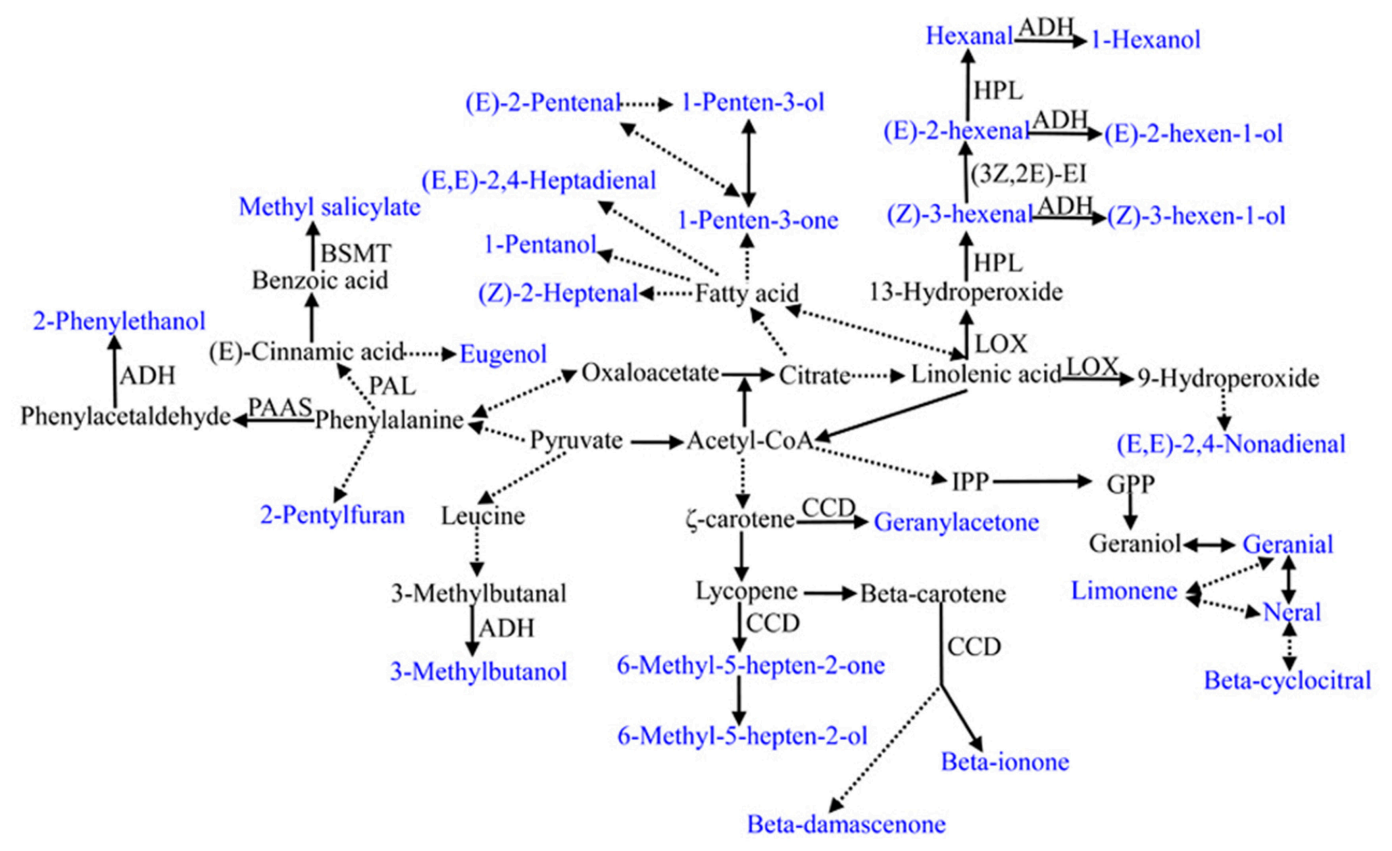
**Summary of metabolic pathways leading to the 28 important flavor-associated volatile synthesis**. Volatiles used in this study are shown in blue. The precursor for 2-isobutylthiazole is not clear and is not listed in this summary. Dashed lines indicate multiple step reactions. Enzymes or genes involved in some volatile synthesis are listed. BSMT, benzoic acid and salicylic acid carboxyl methyltransferase; PAAS, phenylacetaldehyde synthase; PAL, phenylalanine ammonia-lyase; CCD, carotenoid cleavage dioxygenase; LOX, lipoxygenase; ADH, alcohol dehydrogenase; HPL, hydroperoxide lyaser; 3*Z*,2*E*-EI, 3*Z*,2*E*-enal isomerase; IPP, isopentenyl pyrophosphate; GPP, geranyl diphosphate.

Tomato volatiles are mainly derived from four pathways, including the fatty acid, amino acid, terpenoid, and carotenoid pathways. The metabolic pathways of the selected volatiles in this study are shown in Figure 5. All volatiles used in this study are directly or indirectly linked with the tricarboxlic acid cycle (TCA), indicating the fundamental significance of primary metabolism. However, our understanding of the biosynthesis pathways and regulatory networks is only known for a small portion of the most economically significant voaltiles (Klee, 2010). Even for some most important volatiles in tomato, the synthetic pathways have been recently established or remain unknown. For instance, the precursor and the corresponding regulation pathways is unknown for 2-isobutylthiazole, one important sulfur-containing compound in tomato (Iranshahi, 2012). Using significant correlations between traits could be used to build the network structure of the poorly known pathways (Carli et al., 2009).

### Volatile biosynthetic genes

Knowledge of synthetic pathways and the regulatory networks can greatly facilitate the identification of genes encoding biosynthetic enzymes. This can be accomplished by exploiting the whole genomic or expressed sequence databases (Klee, 2010). For instance, Klee and Tieman (2013) reviewed several genes with validated functions in the metabolism of tomato volatiles, including PAR, phenylacetaldehyde reductase; loxC, 13-lipoxygenase; CCD1, carotenoid cleavage dioxygenase; and CXE1, carboxylesterase. LoxC catalyzes the first step in the metabolic pathway that converts 18:2 and 18:3 fatty acids to C6 volatiles, including (Z)-3-hexenal, hexanal, (Z)-3-hexen-1-ol, hexyl alcohol, and hexylacetate (Chen et al., 2004). Volatile terpenoid compounds, including neral, geranial, limonene and beta-cyclocitral, etc. could potentially be derived from carotenoids. These volatiles are all important component of flavor and aroma in tomato (Simkin et al., 2004). *LeCCD1A* (82,184,585–82,195,219 bp) and *LeCCD1B* (82,194,422–82,212,510 bp) are two closely related genes located on chromosome 1 potentially encoding carotenoid cleavage dioxygenases and LeCCD1B. *LeCCD1A* had a great impact on the concentration of beta-ionone and geranylacetone and *LeCCD1B* had a high expression level in ripening fruit (Simkin et al., 2004). In our search, we identified one significant MTA for 6-methyl-5-hepten-2-ol, one important volatile derived from lycopene and another significant MTA for geraylacetone. These two associations were both associated with marker TGS1156. However, the significant level was relatively low and not all carotenoid-derived volatiles were associated with this marker. This could due to the limited markers in this region or the weak marker polymorphic linkage with these two genes. At least five lipoxygenases (TomloxA, TomloxB, TomloxC, TomloxD, and TomloxE) in tomato have been identified. They can greatly impact the generation of C6 aldehyde and alcohol volatiles derived from fatty acids, such as n-hexanal, (Z)-3-hexenal, (E)-2-hexenal, and (Z)-3-hexenol, in both fruit and leaf tissues (Chen et al., 2004). However, researchers have not yet established the biosynthetic pathways for many of the most important volatiles. Here, we selected the 28 most important volatiles in tomato to perform genome-wide association mapping. Our research points to some chromosome regions that may play a significant role in tomato volatile metabolism. However, the marker coverage was relatively small. Combining with the availability of the tomato genome data, and higher density of SSR marker coverage (or SNPs), this research will promote the isolation of novel genes impacting volatiles.

## Conclusions

Phenotypic evaluation on the 28 most important tomato volatiles detected by GC-MS revealed a broad phenotypic variability within diverse accessions across tomato. GWAS between the selected 28 volatiles and 182 SSR markers allowed detection of 125 significant MTAs (*P* < 0.005). We detected at least one MTA for 27 volatiles. Notably, some associations had a very high significant value. For instance, we found a highly significant association between 6-methyl-5-hepten-2-one. This MTA accounted for up to 30% of the phenotypic variation in both year. The other most significant association was detected between (E)-2-hexen-1-ol and SSR287 (Chr2). Some associations were co-localized with previously identified QTLs. We identified several chromosome regions that could greatly impact tomato volatile metabolism. Our results represent a step toward accelerating the rate of flavor related gene discovery.

## Author contributions

JZ and ZZ designed the study. JZ and JTZ carried out the main GC-MS analysis and molecular mapping, analyzed the data, and drafted the manuscript. YL provided the tomato seeds and participated in its design. ML participated in its design and the GC-MS analysis. YX, JL, PC, and FY participated in the phenotype analysis and molecular mapping. All authors corrected and approved the final version.

## Funding

This work was supported by the National Agricultural Science Foundation (No. 201203002), the Program for New Century Excellent Talents in University (No. NCET-12-0474) and National Natural Science Foundation of China (Grant No. 31301498).

## Conflict of interest statement

The authors declare that the research was conducted in the absence of any commercial or financial relationships that could be construed as a potential conflict of interest.
