# Supplementary material for: Genome-Wide Association Mapping for Tomato Volatiles Positively Contributing to Tomato Flavor
Source: Front Plant Sci. 2015 Nov 27;6:1042. doi: 10.3389/fpls.2015.01042 (PMC4661238; doi:10.3389/fpls.2015.01042)
Supplement: Supplementary file 2 [file Table2.DOCX]

***Supplementary Material***

**Genome-wide association mapping for tomato volatiles positively contributing to tomato flavor**

**Jing Zhang^1, 2†^, Jiantao Zhao^1, 2†^, Yao Xu^3^, Jing Liang^4^, Peipei Chang^1^, Fei Yan^1, 2^, Mingjun Li^1^, Yan Liang^1*^, Zhirong Zou^1, 2**^**

^1^College of Horticulture, Northwest A&F University, Shaanxi, State Key Laboratory of Crop Stress Biology for Arid Areas, Yangling 712100, China

^2^Key Laboratory of Protected Horticultural Engineering in Northwest, Ministry of Agriculture, China

^3^College of Forestry, Northwest A&F University, Shaanxi, Yangling 712100, China

^4^Shaanxi Jinpeng Seed Industry Co., Ltd., Yangling 712100, Shaanxi, China

**^†^** These two authors contribute equally to the present study

*** Correspondence:** Yan Liang, College of Horticulture, Northwest A&F University, Shaanxi, State Key Laboratory of Crop Stress Biology for Arid Areas, Yangling 712100, China.

Email: liangyan@nwsuaf.edu.cn

**** Correspondence:** Zhirong Zou, Key Laboratory of Protected Horticultural Engineering in Northwest, College of Horticulture, Northwest A&F University, Litai Street, Yangling, Shaanxi, 712100, China.

Email:zouzhirong2005@hotmail.com

**Table S2** The Pearson correlation coefficients (*r*) among 28 volatiles.

|  | N3O |  |  |  |  |  |  |  |  |  |  |  |  |  |  |  |  |  |  |  |  |  |  |  |  |  |  |  |
| --- | --- | --- | --- | --- | --- | --- | --- | --- | --- | --- | --- | --- | --- | --- | --- | --- | --- | --- | --- | --- | --- | --- | --- | --- | --- | --- | --- | --- |
| MBO | -0.027 | MBO |  |  |  |  |  |  |  |  |  |  |  |  |  |  |  |  |  |  |  |  |  |  |  |  |  |  |
| PTO | -0.106 | 0.233** | PTO |  |  |  |  |  |  |  |  |  |  |  |  |  |  |  |  |  |  |  |  |  |  |  |  |  |
| HXO | -0.196** | 0.133* | 0.215** | HXO |  |  |  |  |  |  |  |  |  |  |  |  |  |  |  |  |  |  |  |  |  |  |  |  |
| X2O | 0.029 | 0.002 | -0.096 | 0.053 | X2O |  |  |  |  |  |  |  |  |  |  |  |  |  |  |  |  |  |  |  |  |  |  |  |
| X3O | 0.011 | 0.260** | 0.176** | 0.156* | -0.052 | X3O |  |  |  |  |  |  |  |  |  |  |  |  |  |  |  |  |  |  |  |  |  |  |
| MHO | 0.125 | 0.024 | -0.021 | -0.146* | 0.121 | 0.132* | MHO |  |  |  |  |  |  |  |  |  |  |  |  |  |  |  |  |  |  |  |  |  |
| PEO | -0.079 | -0.011 | 0.008 | 0.023 | -0.025 | -0.047 | -0.194** | PEO |  |  |  |  |  |  |  |  |  |  |  |  |  |  |  |  |  |  |  |  |
| MSA | 0.042 | -0.132* | -0.260** | -0.167* | 0.143* | -0.163* | -0.007 | 0.131* | MSA |  |  |  |  |  |  |  |  |  |  |  |  |  |  |  |  |  |  |  |
| BIO | -0.093 | -0.096 | -0.143* | -0.137* | 0.068 | -0.008 | -0.025 | 0.043 | 0.132* | BIO |  |  |  |  |  |  |  |  |  |  |  |  |  |  |  |  |  |  |
| N3N | 0.064 | -0.137* | -0.111 | -0.082 | -0.127* | -0.131* | -0.122 | 0.052 | 0.023 | 0.164* | N3N |  |  |  |  |  |  |  |  |  |  |  |  |  |  |  |  |  |
| MHN | -0.019 | 0.085 | 0.253** | 0.05 | -0.06 | -0.056 | -0.07 | -0.058 | -0.084 | -0.136* | -0.12 | MHN |  |  |  |  |  |  |  |  |  |  |  |  |  |  |  |  |
| GYN | -0.052 | 0.03 | 0.155* | 0.017 | -0.029 | -0.036 | 0.002 | -0.073 | -0.114 | -0.073 | 0.051 | 0.149* | GYN |  |  |  |  |  |  |  |  |  |  |  |  |  |  |  |
| EUG | -0.034 | 0.071 | 0.039 | 0.083 | -0.136* | 0.163* | 0.089 | 0.056 | -0.406** | -0.116 | -0.014 | -0.062 | -0.039 | EUG |  |  |  |  |  |  |  |  |  |  |  |  |  |  |
| IBT | -0.052 | -0.016 | -0.023 | -0.028 | -0.022 | 0.107 | 0.001 | 0.013 | -0.109 | -0.047 | -0.05 | 0.127* | -0.001 | -0.038 | IBT |  |  |  |  |  |  |  |  |  |  |  |  |  |
| LMN | 0.150* | 0.012 | -0.171* | -0.178** | 0.044 | -0.159* | 0.003 | 0.095 | 0.078 | 0.129* | 0.136* | -0.032 | -0.045 | -0.03 | -0.137* | LMN |  |  |  |  |  |  |  |  |  |  |  |  |
| PFA | -0.123 | 0.068 | 0.224** | 0.203** | -0.179** | 0.281** | 0.063 | -0.061 | -0.089 | 0.119 | -0.142* | 0.024 | -0.107 | 0.135* | 0.049 | -0.024 | PFA |  |  |  |  |  |  |  |  |  |  |  |
| BCT | 0.005 | 0.179** | 0.464** | 0.249** | 0.041 | 0.044 | -0.089 | -0.119 | -0.165* | -0.288** | -0.137* | 0.350** | 0.231** | -0.045 | 0.046 | -0.093 | 0.162* | BCT |  |  |  |  |  |  |  |  |  |  |
| GRA | 0.109 | 0.130* | 0.323** | 0.085 | 0.014 | 0.029 | -0.056 | -0.044 | -0.119 | -0.227** | -0.09 | 0.432** | 0.329** | -0.017 | -0.001 | -0.034 | 0.013 | 0.445** | GRA |  |  |  |  |  |  |  |  |  |
| NRA | -0.025 | -0.003 | 0.072 | 0.119 | -0.105 | -0.064 | 0.07 | 0.038 | 0.055 | 0.039 | 0.007 | 0.094 | 0.160* | -0.077 | 0.151* | 0.019 | -0.032 | -0.04 | -0.094 | NRA |  |  |  |  |  |  |  |  |
| X3A | 0.028 | 0.052 | 0.224** | 0.027 | -0.018 | 0.241** | 0.082 | -0.013 | -0.187** | -0.102 | -0.056 | -0.01 | -0.012 | 0.164* | 0.075 | -0.045 | 0.309** | 0.293** | 0.129* | -0.153* | X3A |  |  |  |  |  |  |  |
| X2A | -0.047 | 0.01 | 0.082 | -0.001 | -0.052 | 0.125 | 0.028 | -0.09 | -0.133* | -0.11 | 0.045 | 0.009 | -0.003 | -0.077 | 0.041 | 0.126* | 0.04 | 0.092 | 0.04 | 0.009 | 0.095 | X2A |  |  |  |  |  |  |
| HXA | -0.221** | 0.011 | 0.288** | 0.351** | 0.068 | 0.041 | -0.049 | -0.095 | -0.054 | -0.215** | -0.093 | 0.163* | 0.231** | 0.095 | 0.095 | -0.129* | 0.220** | 0.294** | 0.223** | 0.08 | 0.213** | 0.136* | HXA |  |  |  |  |  |
| HPA | -0.163* | 0.170* | 0.417** | 0.312** | 0.136* | 0.443** | 0.014 | -0.026 | -0.266** | -0.073 | -0.179** | 0.101 | 0.088 | 0.203** | 0.097 | -0.165* | 0.287** | 0.292** | 0.250** | -0.084 | 0.390** | 0.177** | 0.375** | HPA |  |  |  |  |
| HTA | 0.156* | 0.111 | -0.133* | -0.015 | 0.216** | -0.121 | 0.128* | -0.049 | 0.151* | 0.09 | 0.075 | 0.051 | -0.005 | -0.128* | 0.014 | 0.014 | -0.193** | 0.026 | 0.004 | 0.063 | -0.148* | -0.009 | -0.026 | -0.071 | HTA |  |  |  |
| NAA | 0.034 | 0.089 | 0.097 | -0.063 | 0.005 | 0.063 | 0.037 | -0.009 | -0.241** | -0.024 | 0.037 | 0.085 | -0.003 | 0.173* | -0.031 | 0.177** | -0.011 | 0.173* | -0.003 | -0.004 | -0.044 | 0.183** | -0.047 | 0.105 | 0.113 | NAA |  |  |
| BDN | 0.131* | -0.064 | -0.052 | -0.156* | 0.09 | -0.094 | -0.008 | 0.07 | 0.013 | -0.001 | 0.140* | 0.019 | 0.038 | -0.041 | -0.002 | 0.123 | -0.096 | 0.001 | 0.161* | 0.057 | 0.037 | 0.052 | -0.085 | -0.031 | 0.085 | 0.013 | BDN |  |
| PEA | 0.216** | -0.047 | -0.151* | -0.105 | 0.018 | -0.091 | 0.152* | -0.013 | 0.121 | 0.048 | 0.201** | -0.167* | 0.036 | -0.026 | -0.123 | -0.029 | -0.133* | -0.114 | -0.071 | 0.058 | -0.135* | -0.013 | -0.053 | -0.174* | 0.207** | -0.081 | 0.088 | PEA |

Note:1-Penten-3-ol, N3O; 3-Methylbutanol, MBO; 1-Pentanol, PTO;1-Hexanol, HXO; (Z)-2-Hexen-1-ol, X2O; (Z)-3-Hexen-1-ol, X3O; 6-methyl-5-Hepten-2-ol, MHO; 2-Phenylethanol, PEO; Methyl salicylate, MSA; Beta-ionone, BIO; 1-Penten-3-one, N3N; 6-methyl-5-Hepten-2-one, MHN; Geranylacetone, GYN; Eugeno, EUG; 2-Isobutylthiazole, IBT; Limonene, LMN; 2-Pentylfuran, PFA; Beta-Cyclocitral, BCT; Geranial, GRA; Neral, NRA; (E)-3-Hexenal, X3A; (E)-2-Hexenal, X2A; Hexanal, HXA; (Z)-2-Heptenal, HPA; (E,E)-2,4-Heptadienal, HTA; (E,E)-2,4-Nonadienal, NAA; Beta-damascenone, BDN; (E)-2-Pentenal, PEA

*, *P*<0.01; **, *P*<0.001
