# Supplementary material for: Genome-Wide Association Mapping for Tomato Volatiles Positively Contributing to Tomato Flavor
Source: Front Plant Sci. 2015 Nov 27;6:1042. doi: 10.3389/fpls.2015.01042 (PMC4661238; doi:10.3389/fpls.2015.01042)
Supplement: Supplementary file 3 [file Table3.DOCX]

***Supplementary Material***

**Genome-wide association mapping for tomato volatiles positively contributing to tomato flavor**

**Jing Zhang^1, 2†^, Jiantao Zhao^1, 2†^, Yao Xu^3^, Jing Liang^4^, Peipei Chang^1^, Fei Yan^1, 2^, Mingjun Li^1^, Yan Liang^1*^, Zhirong Zou^1, 2**^**

^1^College of Horticulture, Northwest A&F University, Shaanxi, State Key Laboratory of Crop Stress Biology for Arid Areas, Yangling 712100, China

^2^Key Laboratory of Protected Horticultural Engineering in Northwest, Ministry of Agriculture, China

^3^College of Forestry, Northwest A&F University, Shaanxi, Yangling 712100, China

^4^Shaanxi Jinpeng Seed Industry Co., Ltd., Yangling 712100, Shaanxi, China

**^†^** These two authors contribute equally to the present study

*** Correspondence:** Yan Liang, College of Horticulture, Northwest A&F University, Shaanxi, State Key Laboratory of Crop Stress Biology for Arid Areas, Yangling 712100, China.

Email: liangyan@nwsuaf.edu.cn

**** Correspondence:** Zhirong Zou, Key Laboratory of Protected Horticultural Engineering in Northwest, College of Horticulture, Northwest A&F University, Litai Street, Yangling, Shaanxi, 712100, China.

Email:zouzhirong2005@hotmail.com

**Table S3** Volatile compounds, structures, identification and their precursors of the 28 most important volatiles.

| Compound | Structure | RI^a^ | RI^b^ | Identificaion^c^ | Precursor |
| --- | --- | --- | --- | --- | --- |
| 1-Penten-3-ol |  | 1165 | 1164 | MS,RI | Fatty acid |
| 3-Methylbutanol |  | 1206 | 1206 | MS,RI | Leucine |
| 1-Pentanol |  | 1254 | 1254 | MS,RI | Fatty acid |
| 1-Hexanol |  | 1350 | 1350 | MS,RI | Fatty acid |
| (E)-2-Hexen-1-ol |  | 1400 | 1400 | MS,RI | Fatty acid |
| (Z)-3-Hexen-1-ol |  | 1365 | 1363 | MS,RI | Fatty acid |
| 6-Methyl-5-hepten-2-ol |  | 1466 | 1466 | MS,RI | Carotenoid |
| 2-Phenylethanol |  | 1933 | 1930 | MS,RI | Phenylalanine |
| Methyl salicylate |  | 1788 | 1788 | MS,RI | Phenylalanine |
| Beta-ionone |  | 1968 | 1968 | MS,RI | Carotenoid |
| 1-Penten-3-one |  | 1054 | 1056 | MS,RI | Fatty acid |
| 6-Methyl-5-hepten-2-one |  | 1340 | 1340 | MS,RI | Carotenoid |
| Geranylacetone |  | 1874 | 1866 | MS,RI | Carotenoid |
| Eugenol |  | 2172 | 2172 | MS,RI | Phenylalanine |
| 2-Isobutylthiazole |  | 1404 | 1404 | MS,RI | Unknown |
| Limonene |  | 1209 | 1209 | MS,RI | Carotenoid |
| 2-Pentylfuran |  | 1231 | 1231 | MS,RI | Phenylalanine |
| Beta-cyclocitral |  | 1638 | 1638 | MS,RI | Carotenoid |
| Geranial |  | 1742 | 1739 | MS,RI | Carotenoid |
| Neral |  | 1698 | 1693 | MS,RI | Carotenoid |
| (Z)-3-Hexenal |  | 1393 | 1393 | MS,RI | Fatty acid |
| (E)-2-Hexenal |  | 1236 | 1233 | MS,RI | Fatty acid |
| Hexanal |  | 1078 | 1083 | MS,RI | Fatty acid |
| (Z)-2-Heptenal |  | 1333 | 1333 | MS,RI | Fatty acid |
| (E,E)-2,4-Heptadienal |  | 1500 | 1497 | MS,RI | Fatty acid |
| (E,E)-2,4-Nonadienal |  | 1718 | 1715 | MS,RI | Fatty acid |
| Beta-damascenone |  | 1835 | 1835 | MS,RI | Carotenoid |
| (E)-2-Pentenal |  | 1134 | 1134 | MS,RI | Fatty acid |
| ^a^Retention index calculated on a HP-INNOWAX capillary column(60m×0.25mm×0.25um) with a homologous series of n-alkanes (C7–C30)  ^b^Retention index on average in NIST Chemistry WebBook (<http://webbook.nist.gov/chemistry>)  ^c^Identification proposal is indicated by the following: MS, identification by comparing EI mass spectrum with Wiley and Nist mass spectral database; RI, identification by comparing NIST Chemistry WebBook. | | | | | |
